# 3D Nanofabrication of High‐Resolution Multilayer Fresnel Zone Plates

**DOI:** 10.1002/advs.201800346

**Published:** 2018-06-05

**Authors:** Umut Tunca Sanli, Chengge Jiao, Margarita Baluktsian, Corinne Grévent, Kersten Hahn, Yi Wang, Vesna Srot, Gunther Richter, Iuliia Bykova, Markus Weigand, Gisela Schütz, Kahraman Keskinbora

**Affiliations:** ^1^ Modern Magnetic Systems Max Planck Institute for Intelligent Systems Stuttgart 70569 Germany; ^2^ Thermo Fisher Scientific 5651 GG Eindhoven The Netherlands; ^3^ Stuttgart Center for Electron Microscopy Max Planck Institute for Solid State Research Stuttgart 70569 Germany

**Keywords:** atomic layer deposition, focused ion beam, fresnel zone plates, nanofabrication, X‐ray optics

## Abstract

Focusing X‐rays to single nanometer dimensions is impeded by the lack of high‐quality, high‐resolution optics. Challenges in fabricating high aspect ratio 3D nanostructures limit the quality and the resolution. Multilayer zone plates target this challenge by offering virtually unlimited and freely selectable aspect ratios. Here, a full‐ceramic zone plate is fabricated via atomic layer deposition of multilayers over optical quality glass fibers and subsequent focused ion beam slicing. The quality of the multilayers is confirmed up to an aspect ratio of 500 with zones as thin as 25 nm. Focusing performance of the fabricated zone plate is tested toward the high‐energy limit of a soft X‐ray scanning transmission microscope, achieving a 15 nm half‐pitch cut‐off resolution. Sources of adverse influences are identified, and effective routes for improving the zone plate performance are elaborated, paving a clear path toward using multilayer zone plates in high‐energy X‐ray microscopy. Finally, a new fabrication concept is introduced for making zone plates with precisely tilted zones, targeting even higher resolutions.

## 1 Introduction

X‐ray microscopy emerged as a very strong tool for natural sciences by providing half‐pitch spatial resolutions of about 10 nm^1^, ^2^, ^3^, ^4^ and high penetration depths combined with structural, chemical, and magnetic contrast.^5^, ^6^, ^7^ The importance of X‐ray microscopy will grow in the near future owing to exciting developments such as the emergence of next generation synchrotron sources,^8^, ^9^, ^10^ new X‐ray free electron lasers (XFEL), high brilliance laboratory X‐ray sources and high harmonic generation sources providing radiation in the extreme ultraviolet (EUV) regime.^11^ However, the trend toward micro‐ and nanosciences has placed stringent requirements on the focusing optics such as having high‐resolution and high focusing efficiency simultaneously as well as high radiation hardness and heat resistance to endure extreme loads provided by highly brilliant beams.^12^

One of the most widely used and successful high‐resolution X‐ray focusing optics is the Fresnel zone plate (FZP). It consists of alternating opaque and transparent coaxial annuli, which form the zones. The spatial Rayleigh resolution of an FZP is defined by, Δδ_Rayleigh_ = 1.22 Δ*r*, where Δ*r* is the width of the outermost zone.^13^ In the EUV and soft X‐ray regime, where wavelengths are relatively large and X‐ray matter interactions are dominated by high absorption, lithographically fabricated FZPs^14^, ^15^, ^16^, ^17^, ^18^ have become the standard optics with full‐pitch resolutions of about 30–40 nm, which are still about 20–30 times greater than the utilized wavelength (1.24 nm for 1000 eV X‐rays), leaving much room for improvement. Higher resolutions down to about 15–9 nm half‐pitch have been reported for FZPs fabricated following unconventional and complex double patterning, zone‐doubling and stacking techniques often correlated with strongly reduced diffraction efficiencies.^1^, ^16^, ^17^, ^19^, ^20^ Typical diffraction efficiencies for standard commercial zone plates at soft X‐rays are about 5–10% but rapidly decrease toward higher energy X‐rays. This is due to the required high optical thicknesses correlated with weaker X‐ray‐matter interactions. Realization of thicker zone plates with similar resolution would require nanofabrication of structures with extremely high aspect ratios. As a consequence, other types of optics such as Kirkpatrick–Baez mirrors (KBM),^21^, ^22^ compound refractive lenses (CRL)^23^, ^24^, ^25^, ^26^ and multilayer‐Laue lenses (MLL)^27^, ^28^, ^29^, ^30^, ^31^ are mostly used in this energy range. High resolution KBMs require a complicated active wavefront correction scheme.^22^ More conventional KBMs, on the other hand, usually have focal spots in the sub‐micrometer size range. Furthermore, KBMs require precise alignment in 6 axes (*X*, *Y*, *Z*, pitch, yaw, roll) on two separate stages making them very bulky and expensive. MLLs arose from the difficulty of fabricating FZPs with tilted zones, which are composed of a set of planar linear‐zone plates in a crossed geometry. MLLs suffer from even more stringent alignment requirements due to the chromatic aberration. Very recently, 2D sub‐10 nm resolution has been reported, reinforcing the potential of multilayer optics.^32^ Nevertheless, the versatility and robust performance of FZPs make them one of the most attractive optics in the complete X‐ray range. Therefore, great efforts are being made to develop hard X‐ray FZPs.^20^, ^33^, ^34^, ^35^, ^36^, ^37^, ^38^

An attractive approach toward realizing high‐resolution hard X‐ray FZPs is to coat a cylindrical substrate of a well‐defined, smooth surface with alternating layers of proper material combinations,^39^ and subsequently slicing a FZP from the deposited substrate to a desired thickness. This is the premise of a four decade old research endeavor on “sputtered‐sliced” or as denoted in this paper “multilayer (ML)” FZPs.^40^ ML‐FZPs offer a variety of advantages besides the virtually unlimited aspect ratios. As monolithic optics, they are easy to align compared to optics with multiple elements such as the in situ stacked FZPs, MLLs, or KBMs. In addition, a single ML‐FZP can deliver high performance over a wide X‐ray energy range without re‐aligning the optic. Furthermore, ML‐FZPs can be made out of chemically inert, high melting point ceramic materials that offer high mechanical strength and good resistance against intense X‐ray pulses, which can benefit intense sources such as XFELs.

Since the first trials in the 1980s,^41^, ^42^ various groups have followed roughly the same approach where a metallic wire core is deposited using an omnidirectional deposition method such as sputtering or pulsed laser deposition while rotating the wire core to mimic a conformal deposition. Owing to a combination of fabrication related imperfections in the zones and suboptimal imaging setups, demonstrated direct imaging resolutions have been much worse than the great potential suggested by indirect experiments.^43^, ^44^

A recent method for producing ML‐FZPs is based on atomic layer deposition (ALD) of multilayers over optical quality glass fibers.^45^ The ALD process allows atomic scale precision in zone thickness and excellent conformality through its sequential, self‐limiting surface reactions that lead to cycle based growth.^46^ The superior conformality of ALD eliminates the need for rotating the glass fiber core and discards possible zone errors related to fiber rotation and directional deposition. A further advantage of ALD ML‐FZPs over lithographic methods is the capability of coating a vast number of substrates conformally, such as centimeter long glass fibers or micropillar arrays as discussed below. A virtually unlimited number of ML‐FZPs can be sliced from the deposited substrate batch. The optical thickness of each ML‐FZP can be chosen freely, allowing the delivery of ML‐FZPs optimized for a wide X‐ray energy range, from a single deposition.

**Figure 1:**
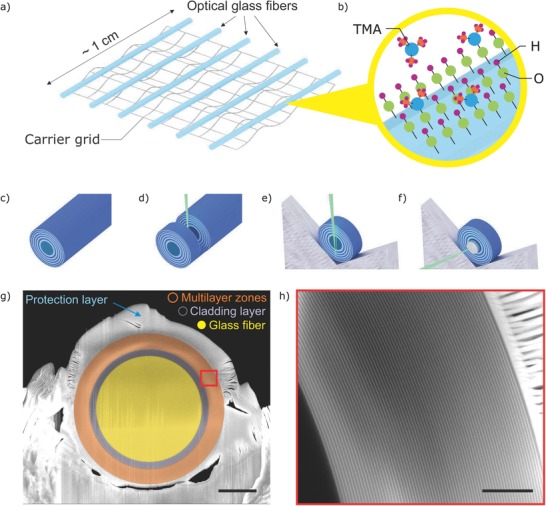
Fabrication stages of ML‐FZP. a) Numerous glass fibers are mounted on a grid. b) Multilayer zones are deposited via ALD. Here, the deposition of the first Al_2_O_3_ layer is depicted by a pulse of trimethylaluminum (TMA) on an OH activated surface. A long deposited fiber c) is sliced d) and mounted on a Mo lift‐out grid e) in the dual beam instrument. f) A beam stopping Pt layer is deposited via ion beam induced deposition in the FIB. g) Scanning electron microscopy (SEM) image of the ML‐FZP mounted on a Mo lift‐out grid. Scale bar is 10 µm. h) SEM image of the multilayer zones defined with the red square of (g). Scale bar is 1 µm.

In this paper, we followed a fully conformal, 3D bottom‐up growth approach based on ALD and subsequent focused ion beam (FIB) slicing (**Figure** **1**). Thus, we realized an Al_2_O_3_–HfO_2_ ML‐FZP of extremely high quality. We investigated its properties by a variety of characterization techniques and a 15 nm half‐pitch cut‐off resolution was recorded via direct imaging experiments in a scanning transmission soft X‐ray microscopy (STXM) set‐up. Encouraged by this success, we introduce a new fabrication concept for making ML‐FZPs with precisely tilted zones. We discuss the implications of tilted FZPs, from coupled wave theory simulations (CWT) and argue why they are crucial for achieving higher resolution FZPs. Finally, the first direct imaging and diffraction efficiency results obtained by our proposed concept are demonstrated.

## 2 Results

### 2.1 Fabrication of High‐Resolution ML‐FZPs

Theoretical diffraction efficiencies of ML‐FZPs consisting of material pairs available to ALD have been calculated to identify best material couples, and were previously published.^39^ Al_2_O_3_–HfO_2_ material couple offers high diffraction efficiencies for both soft and hard X‐rays and therefore were selected as the materials of the ML‐FZP. Another advantage of Al_2_O_3_ and HfO_2_ is their high melting temperatures of 2072 and 2758 °C respectively, providing a high chemical stability.

The Al_2_O_3_–HfO_2_ multilayers were deposited via ALD at 290 °C on several optical quality glass fiber cores (Figure  1a,b), using the parameters detailed in the methods section. Prior to deposition for the ML‐FZP, growth characteristics of the Al_2_O_3_ and HfO_2_ thin films were calibrated by test depositions on Si wafers via a spectroscopic ellipsometry and found to be 0.078 and 0.097 nm per cycle, respectively. A nonstandard 1:2 line (HfO_2_) to space (Al_2_O_3_) ratio was used instead of the standard 1:1. This allowed for a faster deposition due to shorter Al_2_O_3_ deposition cycle (see the Experimental Section).

The deposited glass fibers (Figure  1c) were sliced using a Ga^+^ FIB as demonstrated schematically in Figure  1d. The ML‐FZP was mounted on a Mo lift‐out grid in the dual beam instrument by using a micromanipulator. Prior to FIB slicing a protective layer of Pt was coated over the FZP by FIB induced deposition (FIBID) (Figure  1g).^39^, ^45^ The ML‐FZP was thinned down to an optical thickness of 700 nm via Ga^+^ FIB, which is optimized for the soft X‐ray range. An additional Pt beam‐stop layer of diameter *d* = 25 µm was deposited in the center of the FZP, also by FIBID as illustrated in Figure  1f. The resulting ML‐FZP has a diameter of *d* = 39.4 µm, outermost zone width of Δ*r* = 25 nm, inactive central obstruction of *d*_co_ = 31.4 µm with 4 µm of zone thickness and an aspect ratio of 28. The scanning electron microscopy (SEM) images of the ML‐FZP, prior to FIBID of Pt beam‐stop are shown in Figure  1g,h.

### 2.2 Characterization of ML‐FZP Zones

**Figure 2:**
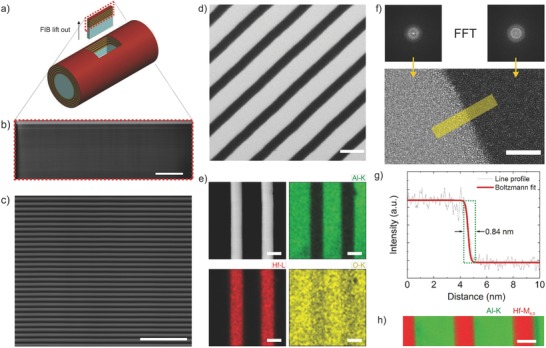
Characterization of multilayer zones along the optical axis. a) Illustration showing location and orientation of the imaged sample. A rectangular prism is lifted out from the deposited fiber by using FIB. b) HAADF image of the lifted out lamella captured using STEM mode in dual beam instrument. The aspect ratio of the structure is larger than 500. Scale bar is 2 µm. c) Higher magnification image of the same lamella shows linear high‐aspect‐ratio multilayer zones. Scale bar is 500 nm. d) STEM bright‐field image of the zones. Scale bar is 50 nm. e) STEM HAADF image and EDX maps of Al–K, Hf–L, and O–K. Scale bars are identical and correspond to 25 nm. f) HRTEM image of the Al_2_O_3_–HfO_2_ interface and FFT confirming fully amorphous zones. Scale bar is 5 nm. g) Intensity line profile of the yellow region of the HRTEM image confirming molecularly sharp interface well below 1 nm. FWHM of the first derivative to the fitted curve is 0.33 nm. h) STEM EELS map of Al–K and Hf–M_4, 5_. Multiple linear least square fitting was used to subtract the background. Scale bar is 20 nm.

To investigate the zone quality in the optical axis, a rectangular lamella was lifted out in the FIB from the deposited glass–fiber as depicted in **Figure** **2**a. This sample was thinned down to about 250 nm for SEM analysis in the transmission mode (STEM), and below 100 nm for high‐resolution transmission electron microscopy (HRTEM) analysis. High‐angle annular dark‐field images (HAADF) of the zones in the STEM mode of an SEM is shown in Figure  2b,c. The multilayer zones keep their linearity and constitute very high structural quality even through a very long sample of 12.6 µm (Figure  2b) reaching an extremely high aspect ratio of above 500. The zones can be seen in higher magnification in Figure  2c–f. The HRTEM of the interface region shows a high mass‐thickness contrast (Figure  2f). Fast Fourier transforms (FFT) prove the multilayers to be amorphous. A line profile of the interface region of Figure  2f shows an interface sharpness on a molecular level (measured to be 0.84 nm), with a full width at half maximum (FWHM) of its first derivative being 0.33 nm at the interface transition (Figure  2g).

The energy‐dispersive X‐ray spectroscopy (EDX) elemental mapping of Figure  2e demonstrates well‐defined, high‐quality zones with abrupt structural and chemical interfaces. With the electron energy‐loss spectroscopy (EELS) map in Figure  2h, presenting the integrated Al‐K and Hf‐M_4,5_ edge intensities, the interfaces of Al_2_O_3_ and HfO_2_ were further confirmed to be chemically abrupt. This is attributed to the chemically stable nature of the amorphous ceramic films deposited via ALD, lacking fast diffusion paths such as grain boundaries. It has been shown that amorphous films, if dense enough, are better diffusion barriers due to lack of grain boundaries.^47^, ^48^, ^49^, ^50^

The chemical composition of the thin films were estimated to be 38 at% Al to 62 at% O for alumina films and 34 at% Hf to 66 at% O for hafnia films by wavelength‐dispersive X‐ray spectroscopy (WDX). Chemical compositions were further confirmed to be Al_2_O_3_ and HfO_2_ by X‐ray photoelectron spectroscopy (XPS) (Figure S1, Supporting Information), EDX (Figure  2e), and EELS (Figure  2h) analysis. The XPS analysis showed very low <1.5 at% C for both Al_2_O_3_ and HfO_2_ thin films. Volumetric mass densities of the thin films were estimated from electron density obtained by X‐ray reflectometry (XRR) analysis and determined to be ρ = 3.0 g cm^−3^ and ρ = 8.9 g cm^−3^ for Al_2_O_3_ and HfO_2_ thin films, respectively (Figures S2 and S3, Supporting Information).

### 2.3 Zone Pattern Inaccuracies

Inaccuracies in the zone pattern may reduce the resolution and diffraction efficiency of the FZP and cause aberrations. The effect of eccentricity, nonconcentric zones, radial displacement of the zones and zone roughness have been discussed elsewhere.^43^, ^51^

The ALD ML‐FZPs with glass fiber cores are expected to have concentric zones (concentricity: γ < 1.2Δ*r*) by nature of the fabrication method. The eccentricity (Δ*R*/*R*) was estimated to be 0.0032 and within the tolerance values^51^ (eccentricity: Δ*R*/*R* < 0.35N^−1^) according to the measurements from the SEM image of Figure  1g. Therefore, astigmatism or coma aberrations are not expected.

However, if the deposition thicknesses are not strictly controlled, the stochastic nature of the bottom‐up fabrication approach^52^ may result in systematic or random radial displacement of the zones,^43^ and induce spherical aberrations. The tolerable radial displacement of the outermost zone has been calculated to be within two outermost zone widths.^51^ Considering an exact core diameter of 30 µm, the measured radial displacement of the outermost zone is 9 nm and well within the tolerable values (<2Δ*r*). However, detailed characterization of the glass fiber cores show that the diameter of different glass fibers vary between 30 and 31.5 µm. The zone plate of interest of this paper has a measured core diameter of 31.4 µm. However the multilayer design was made for a FZP core of 30 µm. This causes a systematic radial displacement of the zones by 700 nm. If the zone thicknesses are accurate, the systematic shift of the zones arising from nonexact diameter of the inactive core, is expected to slightly modify the distribution of the field intensity of the focusing ring.^53^, ^54^ This effect was simulated using Fourier beam propagation method, confirming a negligible alteration in the focal plane intensity distribution (Figure S4, Supporting Information).

### 2.4 Synchrotron Experiments

The diffraction efficiency and resolving capability of the ML‐FZP was tested in the soft X‐ray range at a state‐of‐the‐art STXM, MAXYMUS (UE‐46 PGM‐2) beamline located at the synchrotron facility BESSY II.^55^

**Figure 3:**
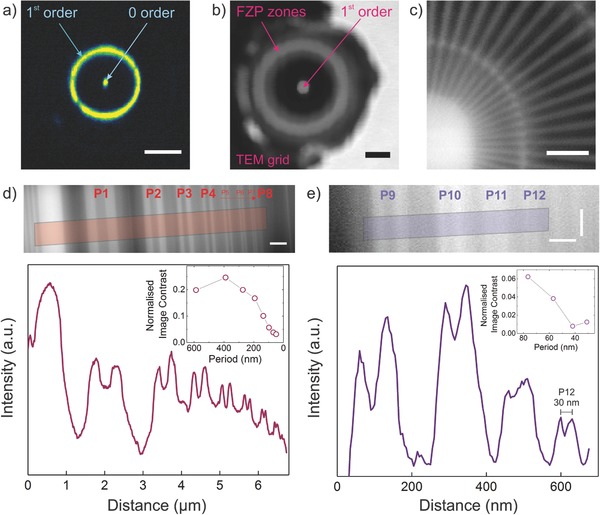
Synchrotron experiments at BESSY II, UE46‐PGM2. a) Charge coupled device (CCD) image of a scintillator screen showing the 1st order diffraction ring. For this image an order selecting aperture was placed between the FZP and CCD. The scintillator screen was placed further away from the focal point and the image on screen was magnified onto the CCD detector. The ML‐FZP tilt was corrected via a tilt stage until a circular first order focus ring was obtained. The presence of the zero order hints a misalignment of the OSA. The scale bar is 250 nm^−1^. b) Pinhole scan over the FZP to measure the diffraction efficiency. The transmitted light is collected by an avalanche photo diode (APD). Dwell time 2 ms. Step size 500 nm × 500 nm. Photon energy 1400 eV. Scale bar is 10 µm. c) STXM image of the Siemens Star test pattern. The 30 nm features of the innermost ring are resolved. Dwell time 10 ms. Energy 1198 eV. Step size 10 × 10 nm. Scale bar is 500 nm. d) STXM image of P1 to P8 of the BAM L‐200 test structure (top) and its integrated intensity profile and normalized Michelson image contrast (bottom graph). All features P1 (587 nm)–P8 (48.5 nm) are resolved. Dwell time 10 ms. Step size 10 × 10 nm. Photon energy 1200 eV. Scale bar is 500 nm. e) STXM image of the P9 (76.5 nm) to P12 (30 nm) of the BAM L‐200 test structure (top) and its integrated (15 pixels wide) intensity profile and normalized Michelson image contrast (bottom graph). 30 nm full period structure (P12) is resolved corresponding to 15 nm half‐pitch cut‐off resolution. Dwell time 30 ms. Step size 4 × 5 nm. Photon energy is 1296 eV. Scale bars correspond to horizontal 100 nm and vertical 120 nm.

Prior to imaging experiments, the ML‐FZP was aligned in pitch and yaw using an in‐house constructed tilt stage until a full 1st order diffraction ring was achieved. **Figure** **3**a shows the image of the diffraction ring after alignment. A pinhole scan shows the incident illumination and the 1st order intensity (Figure  3b).The features of a Siemens Star test sample (Zeiss, X30‐30‐2Au) was imaged and the innermost 30 nm structures were resolved clearly at 1198 eV photon energy. This is shown in Figure  3c. To check the ultimate imaging resolution another test sample with smaller features (BAM, L‐200) was used. The STXM results are presented in Figure  3d,e. The 30 nm full period structure (P12), corresponding to a 15 nm half‐pitch, was resolved (cut‐off) in Figure  3e. To the best of our knowledge, this is the highest imaging resolution obtained by using a ML‐FZP.

It is known that the presence of a central obstruction causes the intensity in the focal spot to shift to side lobes.^56^, ^57^ This has two major effects for high‐resolution FZPs. It causes a halo effect around the imaged structures, and the modulation transfer function increases at high frequencies just before the cut‐off resolution.^43^ These effects can be seen in Figure  3d and e, respectively. The effects arising from large central obstruction would be significantly reduced by increasing the deposition thickness.^43^

The diffraction efficiency measurements were conducted via scanning a pinhole over the FZP (Figure  3b) at various X‐ray energies. The method for diffraction efficiency estimation has been discussed elsewhere.^58^ The measured diffraction efficiencies were found to be lower than ideal. A comparison between the measured and calculated values is shown in **Table** **1**. This can be attributed to several independent sources: i) a slight imperfection in the zone positions, ii) parasitic deposition of a platinum–gallium–carbon (Pt–Ga–C) layer over the zones during deposition of the beam‐stop via FIBID (Figure S5, Supporting Information), iii) possible thickness variation of the ML‐FZP in the optical axis, and iv) curtaining effects during FIB slicing and polishing. The diffraction efficiency calculations were done according to CWT^59^ using X‐ray interaction data of Henke et al.^60^ The thickness of parasitic Pt–Ga–C layer on the zones was estimated in a separate study to be 115 nm (Figure S5, Supporting Information). In Table  1 the effect of parasitic Pt–Ga–C deposition on diffraction efficiency is included in a separate column named as “expected D.E.” The atomic percentages of Pt, Ga, and C of FIBID were taken from the values stated in the literature.^61^ The density of the Pt–Ga–C was estimated to be 12.34 g cm^−3^ from weighted mean mass density of the elements. The CWT calculations were done for the outermost period locally. Interdiffusion and roughness were neglected in accordance with the HRTEM, TEM‐EDX, and EELS results. For the calculation, the following parameters were considered: Outermost zone width Δ*r* = 25 nm, densities of ρ = 8.9 g cm^−3^ and ρ = 3.0 g cm^−3^ (Figures S2 and S3 of the Supporting Information for XRR data) and line to space ratio of 1:2 for HfO_2_ and Al_2_O_3_ thin films, respectively. To be able to compare measured and expected diffraction efficiencies a constant optical thickness of 700 nm was considered for the calculation of expected diffraction efficiencies in the soft X‐ray range. The expected performance of the optic in hard X‐rays is included in the Table  1 for 9.0 and 14.4 keV X‐rays. Ideal optical thicknesses were considered for calculating the diffraction efficiencies at the same energies.

**Table 1:** Comparison of measured, expected and ideal diffraction efficiencies at various X‐ray energies. The calculations are done according to CWT (see text and Table S1 in the Supporting Information). The ideal D.E. is the diffraction efficiency for an FZP of ideal design and neglects any Pt–Ga–C spill‐over deposition during beamstop deposition. The X‐ray absorptive effect of spill‐over Pt–Ga–C deposition of thickness 115 nm on FZP zones is included for expected diffraction efficiency values. The theoretical efficiencies increase after the absorption edge

Energy [keV]	Measured D.E. [%]	Expected D.E. [%]	Ideal D.E. [%]
1.4	1.5	2.8	7.4
1.5	1.9	2.4	7.1
1.6a)	0.2	0.7	1.9
9.0	Not measured	26.4	26.9
14.4	Not measured	31.0	31.6

^a)^ The efficiency decrease at 1.6 keV is related to Al–K edge at 1559 eV.

## 3 Discussion

A ML Al_2_O_3_–HfO_2_ FZP with an outermost zone width of Δ*r* = 25 nm, a diameter of *d* = 39.4 µm and a deposition thickness of 4 µm was successfully fabricated. ALD multilayers constituting the zones exhibited chemically abrupt and structurally sharp interfaces on the molecular level. The ML‐FZP focusing performance was studied in detail in the soft X‐ray range at a state of the art STXM (UE46‐PGM2) located at the synchrotron radiation facility BESSY II. The own built soft X‐ray microscope was chosen as the experimental set‐up due to its versatility and stability allowing for highly precise characterization of the focusing performance. The imaging tests revealed a half‐pitch cut‐off resolution of 15 nm for the first time for a ML‐FZP. This is a marked increase over previous related research.^62^ The diffraction efficiency was measured and found to have a peak of 1.9% at 1500 eV, which is about 80% of the theoretically expected value of 2.4%. The undesired spillover deposition of a Pt–Ga–C layer over the zones is identified as a major reason for the lower than ideal efficiency, especially at lower energies due to increased absorption of this layer. Other reasons could be related to curtaining effects during FIB polishing and imperfections during the ALD or FIB slicing process.

**Figure 4:**
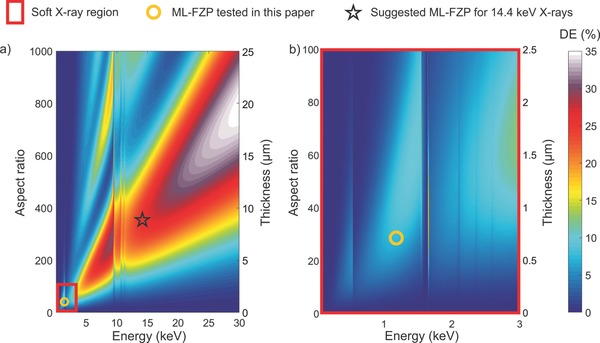
Diffraction efficiency maps of Al_2_O_3_–HfO_2_ ML‐FZP. Calculations were done according to thin grating approximation as a function of aspect ratio, optical thickness and X‐ray energy for Δ*r* = 25 nm. a) Diffraction efficiency map from 100 eV to 30 keV. b) Diffraction efficiency map of the region marked in red in (a). The corresponding numbers to color coding represents the diffraction efficiency in percent.

The diffraction efficiency maps as a function of energy and zone plate thickness were calculated for up to 30 keV using the thin grating approximation^63^ and depicted in **Figure** **4**a,b. The alumina‐hafnia ML‐FZP exhibit reasonably high diffraction efficiencies in the soft X‐ray range. Nevertheless, ML‐FZPs certainly excel at the hard X‐ray regime with strongly increased diffraction efficiencies for even higher energies. The expected efficiencies are as high as 31% for 14.4 keV X‐rays for a ML‐FZP of optical thickness 10.2 µm (Table  1), an energy relevant for Mössbauer spectroscopy experiments. A structure with such high optical thickness is successfully prepared (Figure  2b). This optic offers a combination of high‐resolution and high diffraction efficiency at a very desired X‐ray energy.

One concern in the X‐ray optics community is that sputter‐sliced FZPs in general, tend to have zones with high roughness and low circularity, which has been shown to be detrimental to the imaging capabilities.^43^ Instead, when deposited via ALD on optical quality glass fibers, zones with low roughness both radially and longitudinally have been confirmed in this research with in‐depth electron microscopy analyses of structures over an aspect ratio of 500 (Figure  2b). This proves the potential of ALD fabricated ML‐FZPs for the hard X‐ray regime. Furthermore, we have shown via SEM measurements that the circularity of the substrate and resulting multilayer stack is within the tolerances.

The high melting temperatures and radiation resistance of Al_2_O_3_ (m.p. = 2072 °C) and HfO_2_ (m.p. = 2758 °C) layers are desirable properties for withstanding intense X‐ray pulses of XFELs and emerging diffraction limited synchrotrons. Furthermore, the relatively low X‐ray absorption of Al_2_O_3_ and HfO_2_ would result in less heating during intense X‐ray pulses, improving their life‐time.

The electron microscopy analyses confirm the zones to be fully amorphous. The advantage of amorphous layers is two‐fold. First, amorphous layers eliminate the possibility of undesired scattering from the crystallites, particularly important for hard X‐ray focusing. Second, the lack of grain boundaries ensure that there are no fast diffusion paths available which could lead to interdiffusion between the layers and decrease the performance.^59^ However, any possible change of the microstructure of the zones under extremely intense X‐ray radiation, such as XFEL radiation was not tested and should be addressed in a future study.

A current disadvantage of the method that the diameters being small due to the slow deposition rates, can be overcome by using spatial ALD, that allows for deposition speeds up to 3.6 µm h^−1^,^64^ which is about 2 orders of magnitude faster than the process utilized in this paper. The overall fabrication time would be decreased by faster FIB slicing of the ML‐FZP from the deposit by using recently developed, commercially available FIBs with higher beam currents, multiply charged ion species, or heavier ions.

### 3.1 The Future of Multilayer Fresnel Zone Plates

**Figure 5:**
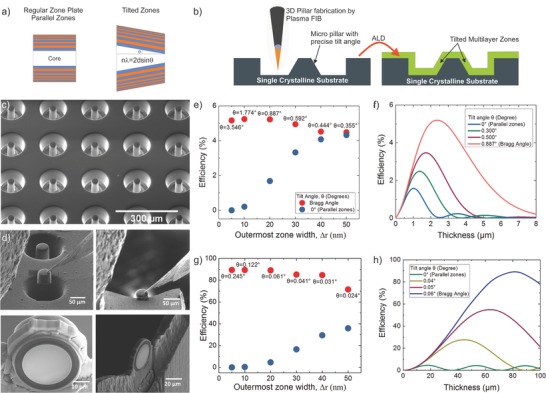
a) In tilted ML‐FZP the zones are tilted with respect to the optical axis. The peak efficiency is achieved if the zones are tilted to the Bragg angle. The concept of regular ML‐FZP with parallel zones and the suggested ML‐FZP with tilted zones is sketched in a side view. b) The fabrication steps of tilted ML‐FZPs is illustrated. c) An SEM image of the tapered micropillar array fabricated via Plasma Focused Ion Beam (PFIB). Multilayer zones of Al_2_O_3_–SiO_2_ are deposited on the tilted micropillar array using ALD. d) A planar liftout strategy is followed to prepare tilted ML‐FZPs from the deposited array. Individual tilted ML‐FZPs are then mounted on Mo lift‐out grids similar to regular ML‐FZPs. e) Calculated diffraction efficiencies of ML‐FZPs at their optimum optical thickness having parallel and tilted zones as a function of outermost zone width, Δ*r* for 1 keV X‐rays. f) Calculated diffraction efficiency of an Al_2_O_3_–SiO_2_ ML‐FZP of Δ*r* = 20 nm for 1 keV X‐rays as a function of tilt angle, θ. g) Calculated diffraction efficiencies of ML‐FZPs at their optimum optical thickness having parallel and tilted zones as a function of outermost zone width, Δ*r* for 14.4 keV X‐rays. h) Calculated diffraction efficiency of an Al_2_O_3_–SiO_2_ ML‐FZP of Δ*r* = 20 nm for 14.4 keV X‐rays as a function of tilt angle, θ. All the efficiencies are calculated according to CWT locally, considering only the outermost period and not integrated to the FZP area.

It is known that FZPs with zones that are parallel to the optical axis come with extreme penalties to the focusing efficiencies at small Δ*r* (**Figure** **5**e–h) due to wave coupling effects.^59^, ^65^ This negates one of the main advantages of fabricating FZPs via thin film deposition techniques, namely, the possibility to deposit extremely fine outermost zones for high resolution optics. Proposed solutions to this particular problem include stacking of binary FZPs to create tilted zones,^18^ multilayer Laue lens pairs,^27^, ^28^, ^29^, ^30^, ^66^ and depositing on drawn tapered fibers.^67^, ^68^ Stacking is interesting but is composed of several subsequent lithography steps, which increases the chances of fabrication errors and is ultimately limited by the number of lithography steps one can afford. MLLs have recently drawn immense interest but having monolithic optics is more desirable as it decreases the complexity of the imaging setup. Depositing on drawn fibers often raises questions. Does the fiber stay circular when drawn? How well can the tapering angle be controlled? These questions need to be addressed at the cost of labor intensive characterization and is difficult to make sure each fiber satisfies the necessary criteria. Here, we propose and demonstrate a new concept based on improvements in the focused ion beam technology that resolves the above mentioned issues. The method is depicted in Figure  5a,b. In this method a Xe^+^ plasma FIB is used to microfabricate arrays of tapered pillars (Figure  5c) with controlled slanting angles. Here, we used a single crystal gold substrate with (111) orientation in order to maximize the PFIB sputter yield and minimize the fabrication time. Then, an alumina/silica multilayer structure with an outermost zone width of 20 nm was deposited using ALD (see the Experimental Section). In Figure  5d, the plan‐view lift out process of the ML‐FZP with tilted zones, is depicted. The tilted ML‐FZP is transferred on a TEM sample holder as described before and flipped 90° prior to the final polishing step. After the final polishing, the ML‐FZP with tilted zones is shown ready for use (Figure  5d).

The theoretical local diffraction efficiency of the outermost zone period of the suggested alumina/silica ML‐FZP is shown in Figure  5e–h. The change in diffraction efficiency for parallel and tilted zones as a function of outermost zone width, Δ*r* is shown in Figure  5e for 1 keV X‐rays, and Figure  5g for 14.4 keV X‐rays. Figure  5f,h shows that the diffraction efficiencies have a maximum for the Bragg angle. However, even in the case of a nonperfect tilting angle, significant diffraction efficiency gains are possible. This is important because with the suggested fabrication method, it is not possible to bring each zone to its local Bragg angle as Bragg angle changes slightly for every zone as each has a slightly different thickness. The tapered pillars were formed essentially by polishing the sidewalls by a focused beam of Xe^+^ ions resulting in a smooth sidewall profile while the tilt angle can be easily controlled by varying the process parameters. The milling strategy and process parameters are discussed in the Supporting Information (Figures S6 and S7, Supporting Information). Using gold as the central obstruction makes the need of depositing a beamstop by means of FIBID obsolete, hence avoids the complications discussed in the first part of this work.

**Figure 6:**
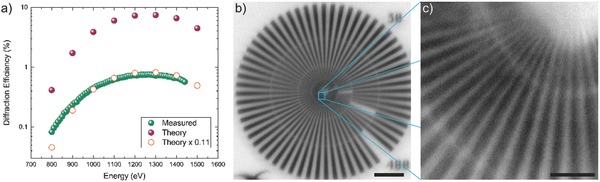
a) Measured (green spheres) versus theoretical diffraction efficiencies (purple spheres and orange circles) for the tilted ML‐FZPs. The theoretical calculations were done according to CWT. The difference of the thickness of each period was taken into account (nonlocal, integrated). b) STXM image of the Siemens Star test sample. Energy is 1175 eV. Step size is 20 nm. Scale bar is 5 µm. c) STXM image of a quarter of the inner rings of the Siemens Star test sample. Energy is 1175 eV. Step size is 10 nm. Scale bar is 500 nm.

The performance of a tilted ML‐FZP was tested also at the MAXYMUS (UE‐46 PGM‐2) beamline. The measured diffraction efficiency of the tilted ML‐FZP achieved 11% of the theoretically expected value in an X‐ray energy range between 800 and 1450 eV (**Figure** **6**a), and it is in good qualitative agreement with the theory. An overall (Figure  6b) and close up (Figure  6c) image of the Siemens Star test sample shows that 30 nm structures were clearly resolved. This very first successful imaging experiments promise higher efficiencies and resolutions through process optimization in the near future.

## 4 Conclusions

Preparation of ML‐FZPs with extraordinary zone quality via ALD of amorphous–ceramic multilayers over optical glass fibers and subsequent FIB slicing was demonstrated. Consequently, the ML‐FZPs achieved up to 80% of their theoretical efficiency and enabled 15 nm half‐pitch cut‐off resolution in direct imaging experiments, which is remarkably close to the theoretical predictions. We also introduced a new fabrication strategy, which allows the fabrication of ML‐FZPs with precisely tilted zones. To the best of our knowledge, this is the first demonstration of a manufacturing route that delivers FZPs with precise tilt‐angles. Furthermore, we demonstrated that the proposed fabrication concept enables the tilting angle to be tailored arbitrarily, and enables fabrication of FZPs with different tilt angles simultaneously. We have shown the first imaging and efficiency analysis using the tilted ML‐FZPs and resolved the smallest 30 nm features of the Siemens Star test sample. Based on the presented experimental results and calculations, it is expected that single nanometer resolutions with high‐diffraction‐efficiencies will soon be available in the soft and hard X‐ray regime with the atomic layer deposited, ceramic ML‐FZPs.

## 5 Experimental Section

*Atomic Layer Deposition*: Optical quality glass fibers from Schott AG (Germany) with the product code A2/30 µm were used as the core. The Al_2_O_3_–HfO_2_ thin films were deposited with a SENTECH SI ALD LL instrument (Germany) at 290 °C. For the Al_2_O_3_ deposition trimethylaluminum (TMA) (99.999+% Al) and deionized H_2_O precursors were used. For the HfO_2_ deposition Hf[N(CH_3_)_2_]_4_ (98+%) and deionized H_2_O precursors were used. Only Hf[N(CH_3_)_2_]_4_ was heated to 70 °C to achieve higher vapor pressure. N_2_ gas of purity 6.0 was used as carrier gas for the precursors. Prior to FZP deposition, growth per cycles (GPC) of the Al_2_O_3_ and HfO_2_ thin films were measured in a test deposition on Si Wafers manufactured via a spectroscopic ellipsometry and determined to be 0.078 and 0.097 nm per cycle, respectively. For the ML‐FZP nonstandard 1:2 line (HfO_2_) to space (Al_2_O_3_) ratio was used instead of the standard 1:1, that allowed for faster deposition. The N_2_ flow, chamber pressures and pulse and purge times are listed in **Table** **2**.

**Table 2:** List of ALD Parameters of Al_2_O_3_ and HfO_2_ zones for the ML‐FZP fabrication

	Precursor	N_2_ flow [sccm]	t_Pulse_ [ms]	*t*_Purge_ [ms]	Pressure [Pa]
Al_2_O_3_	TMA	80	20	1980	6.3
	H_2_O	80	20	1980	
HfO_2_	TDMAHf	40	40	15 000	10.7
	H_2_O	40	20	10 000	

*ALD for Tapered ML‐FZPs*: Al_2_O_3_–SiO_2_ multilayers of 1:1 line to space ratio were deposited on tapered micropillars prepared by PFIB (see Micropillar Fabrication via PFIB) using a SENTECH SI ALD LL instrument (Germany) at 200 °C. For the Al_2_O_3_ deposition TMA (99.999+% Al) and deionized H_2_O precursors were used. For the SiO_2_ deposition bis[diethylamino]silane (BDEAS) and O_2_ plasma (O_2_ gas of purity 6.0) were used. The plasma power was 200 W. Only BDEAS was heated to 70 °C to achieve the necessary vapor pressure. N_2_ gas of purity 6.0 was used as carrier gas for the precursors. Prior to FZP deposition, GPC of the Al_2_O_3_ and SiO_2_ thin films were measured in a test deposition on Si Wafers via a spectroscopic ellipsometry and determined to be 0.059 and 0.1304 nm per cycle, respectively. The N_2_ flow, chamber pressures and pulse and purge times are listed in **Table** **3**.

**Table 3:** List of ALD parameters for Al_2_O_3_ and SiO_2_ zones for the tilted ML‐FZP fabrication

	Precursor	N_2_ flow [sccm]	*t*_Pulse_ [ms]	*t*_Purge_ [ms]	Pressure [Pa]
Al_2_O_3_	TMA	80	20	1980	6.3
	H_2_O	80	20	1980	
SiO_2_	BDEAS	40	140	1860	20
	O_2_ plasma	200	1000	1000	

*FIB Slicing, Transfer, and Polishing of the ML‐FZP*: The deposited glass fiber was sliced and polished with a Ga^+^ ion beam in the FEI Nova NanoLab 600 dual‐beam instrument. The transfer of the multilayer slice on to a Mo lift‐out grid was performed by a micromanipulator in the dual‐beam instrument. A comment on FIB induced damage can be found in the Supporting Information.

*Micropillar Fabrication via PFIB*: Tapered micropillars were fabricated from single crystalline samples of Si(100) and Au(111) (purity 99.999% from MaTecK Material‐Technologie and Kristalle GmbH) by using a ThermoScientific Helios Plasma FIB. iFast recipes were created for controlling the pillar milling processes automatically. In order to fabricate pillars with reasonable throughput, high FIB beam currents for milling are selected. To achieve a lower taper angle of the pillars, multiple concentric angular milling ring patterns were generated by iFast recipe with outer diameters dynamically reducing in a pre‐defined step size during the pillar fabrications. The fabrication parameters are summarized in **Table** **4**. The standard deviation in the tilt angle for a 5 × 5 array is less than 0.1°. SEM images of the fabricated pillars are shown in the Supporting Information.

**Table 4:** Pillar fabrication parameters for Si(100) and Au(111) substrates for various tilt angles. In the marked steps the diameter of the milling area was reduced dynamically with a defined step size

Substrate	Mill Step 1	Mill Step 2	Mill Step 3	Tilt angle [°]	Mill time [min]
Si(100)	1.3 µA	n/a	n/a	9	32
Si(100)	1.3 µA	470 nA	n/a	5	38
Si(100)	1.3 µA	59 nA*, 6 µm step‐size for outer ring reducing	n/a	1	50
Si(100)	1.3 µA	59 nA*, 2 µm step‐size for outer ring reducing	n/a	0.85	60
Au(111)	1.3 µA	180 nA*, 2 µm step‐size for outer ring reducing	n/a	1	20
Au(111)	1.3 µA	59 nA*, 2 µm step‐size for outer ring reducing	15 nA*, 1.5 µm step‐size for outer ring reducing	0.8	29

*Wavelength‐Dispersive X‐Ray Spectroscopy*: Al_2_O_3_ and HfO_2_ thin films were deposited via ALD on separate 4 in. Si wafers using the ALD parameters of Table  2. Wavelength‐dispersive X‐ray spectroscopy was performed with a Cameca SX100 Electron Probe Micro Analyzer. Al_2_O_3_ and HfO_2_ thin films were measured using an accelerating voltage of 5 kV, a beam current of 40 nA and a beam diameter of 5 µm. To eliminate the instrument effects an analysis with matched standards was performed. A pure sapphire mineral was used as a standard for the elements Al and O, and a Hf crystal was used as a standard for the element Hf. A background correction was done by measuring the mean intensity of the background radiation on both sides of the peak and subtracting this value from the intensity of the element characteristic X‐rays. To account for the matrix effects, a *ZAF* (*Z*: atomic number, *A*: absorption, *F*: fluorescence excitation) correction was done in the WDX software. An iterative procedure was followed using the K‐ratios for calculating the stoichiometry.

*X‐Ray Photoelectron Spectroscopy*: Al_2_O_3_ and HfO_2_ thin films were deposited *via* ALD on separate 4 in. Si wafers using the ALD parameters in the Table  2. XPS analysis was performed via a Thermo VG Thetaprobe 300 system with monochromatic Al K_α_ radiation (*hv* = 1468.68 eV; spot size 400 µm). Both samples suffered from high C concentration related to surface contaminants. To confirm, the XPS measurements were repeated after an in situ Ar^+^ sputter‐etching (2 keV, 30 s, raster 3 × 3 mm). The XPS analysis with in situ Ar^+^ sputter‐etching showed <1.5 at% C concentration. The very low C1s peak present at the XPS data of Figure S1b,f (Supporting Information) are related to the surface C–C and C–H contamination during the XPS measurement, which was not visible directly after the Ar^+^ sputter‐etching. This suggests that the C content in the Al_2_O_3_ and HfO_2_ films should be well below <1.5 at%.

*Scanning‐ and High‐Resolution Transmission Electron Microscopy*: A cross section specimen from a deposited glass–fiber in longitudinal direction (Figure  2a) was thinned down to an end thickness of about 100 nm via a FEI Nanolab 600 Ga^+^ dual‐beam instrument. The EELS and EDX measurements were performed on a JEOL JEM‐ARM200F microscope equipped with a cold field‐emission electron source, a DCOR probe corrector (CEOS Co. Ltd.), a 100 mm^2^ JEOL Centurio EDX detector and a Gatan GIF Quantum ERS spectrometer. The microscope was operated at 200 kV, a semiconvergence angle of 21 mrad, giving rise to a probe size of 1 Å for analytical measurements. A collection semiangle of 112 mrad was used for EELS measurements.

HRTEM experiments were performed at 200 kV with JEOL JEM‐ARM200F, equipped with a cold field‐emission gun and a CETCOR image corrector (CEOS Co. Ltd.).

*X‐Ray Reflectometry*: X‐ray reflectometry measurements were conducted with a Siemens D5005 diffractometer equipped with an X‐ray mirror for Cu K_α_ radiation on the same samples explained in XPS section. The data were fitted with the LEPTOS (Bruker) software.

*Synchrotron Measurements*: A STXM beamline (MAXYMUS) with elliptical undulator of the APPLE II type (UE46‐PGMII) located at BESSY II was utilized for the synchrotron experiments. For the diffraction efficiency measurements a pinhole of diameter, *d* = 4 µm was scanned over an area of 85 × 60 µm to image the ML‐FZP mounted on the lift‐out grid as in Figure  3b for each energy. The diffraction efficiency was calculated by dividing average intensity of the 1st order focus to the total intensity on the FZP zones.^58^ For imaging an OSA of 15 µm width was placed between the ML‐FZP and the sample.

Michelson contrast was used for image contrast determination of the BAM sample in Figure  3d,e. For both of the images, the contrast was normalized to the highest (Michelson) contrast region of the Figure  3d (the peak and minimum left to the feature P1). A background subtraction was done in the intensity profile of the Figure  3e.

## Conflict of Interest

The MPG filed a patent application for the proposed techniques. One of the authors, C.J. works for Thermo Fisher Scientific, which produces the Xe^+^ and Ga^+^ plasma FIB instruments used in this work.

## Supporting information

Supplementary
