# Supplementary material for: 3D Nanofabrication of High‐Resolution Multilayer Fresnel Zone Plates
Source: Adv Sci (Weinh). 2018 Jun 5;5(9):1800346. doi: 10.1002/advs.201800346 (PMC6145245; doi:10.1002/advs.201800346)
Supplement: Supplementary file 1 — Supplementary [file ADVS-5-1800346-s001.pdf]

Copyright WILEY-VCH Verlag GmbH & Co. KGaA, 69469 Weinheim, Germany, 2018.

ADVANCED SCIENCE
Open Access

Supporting Information

for *Adv. Sci.,* DOI: 10.1002/advs.201800346

3D Nanofabrication of High-Resolution Multilayer Fresnel Zone Plates

Umut Tunca Sanli, Chengge Jiao, Margarita Baluktsian, Corinne Grévent, Kersten Hahn, Yi Wang, Vesna Srot, Gunther Richter, Iuliia Bykova, Markus Weigand, Gisela Schütz, and Kahraman Keskinbora**

Supporting Information

Umut Tunca Sanli*, Chengge Jiao, Margarita Baluktsian, Corinne Grevent, Kersten Hahn, Yi Wang, Vesna Srot, Gunther Richter, Iuliia Bykova, Markus Weigand, Gisela Schütz, Kahraman Keskinbora*

S.1 X-ray photoelectron spectroscopy (XPS) Spectra

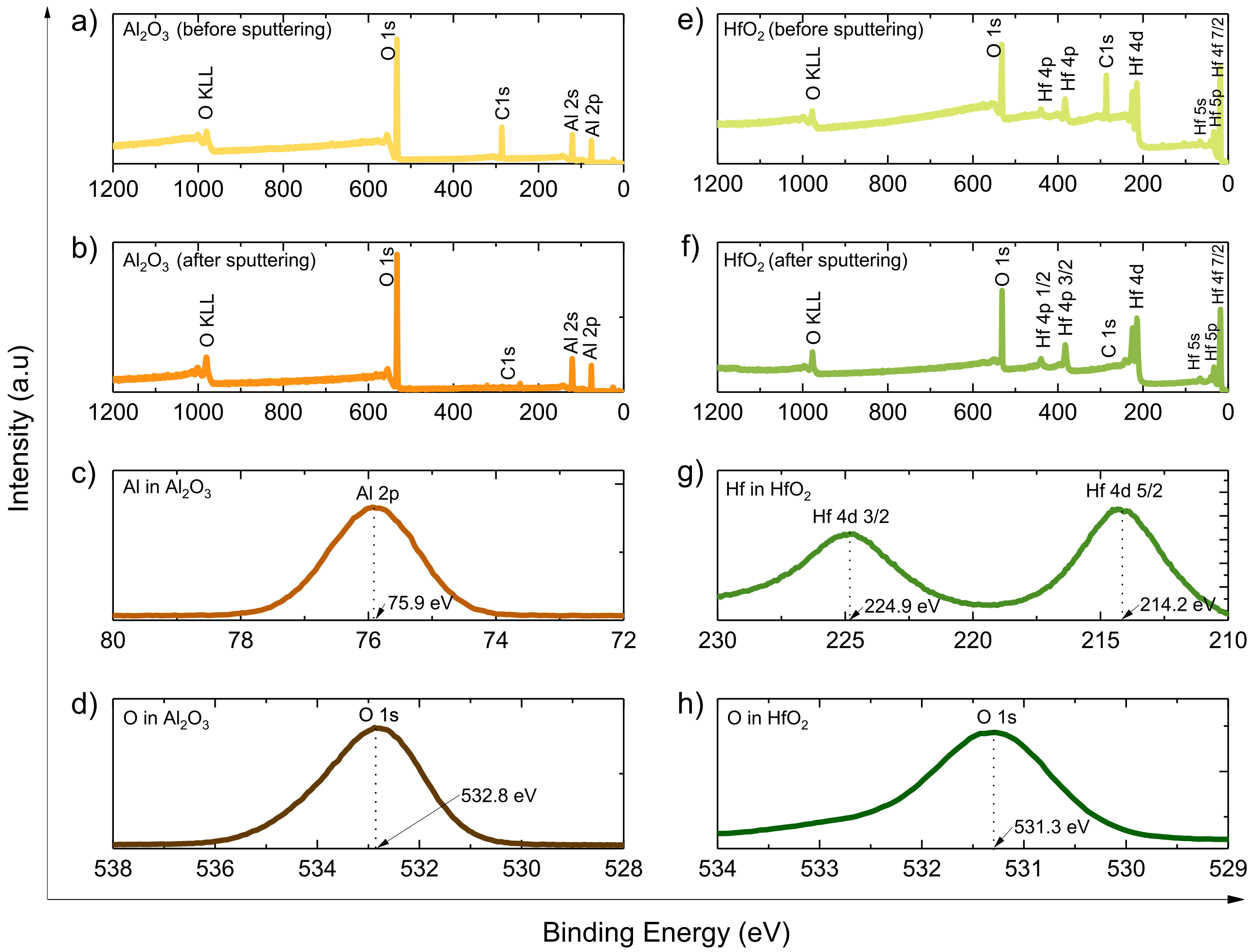

Figure S1: XPS spectra of Al_2O_3 and HfO_2 thin films fabricated via ALD on c-Si Wafer. a) Overall spectral graph of Al_2O_3 before Ar^+ sputtering. b) Overall spectral graph of Al_2O_3 after Ar^+ sputtering to eliminate surface contaminants. The C peak is not visible. c) Al 2p peak of the Al_2O_3 spectra. d) O 1s peak of the Al_2O_3 spectra. e) Overall spectral graph of HfO_2 before Ar^+ sputtering. f) Overall spectral graph of HfO_2 after Ar^+ sputtering to eliminate surface contaminants. The C peak is not visible. g) Hf 4d peak of HfO_2 spectra. h) O 1s peak of the HfO_2 spectra.

S.2 X-ray reflectometry (XRR) spectra and fitting curves

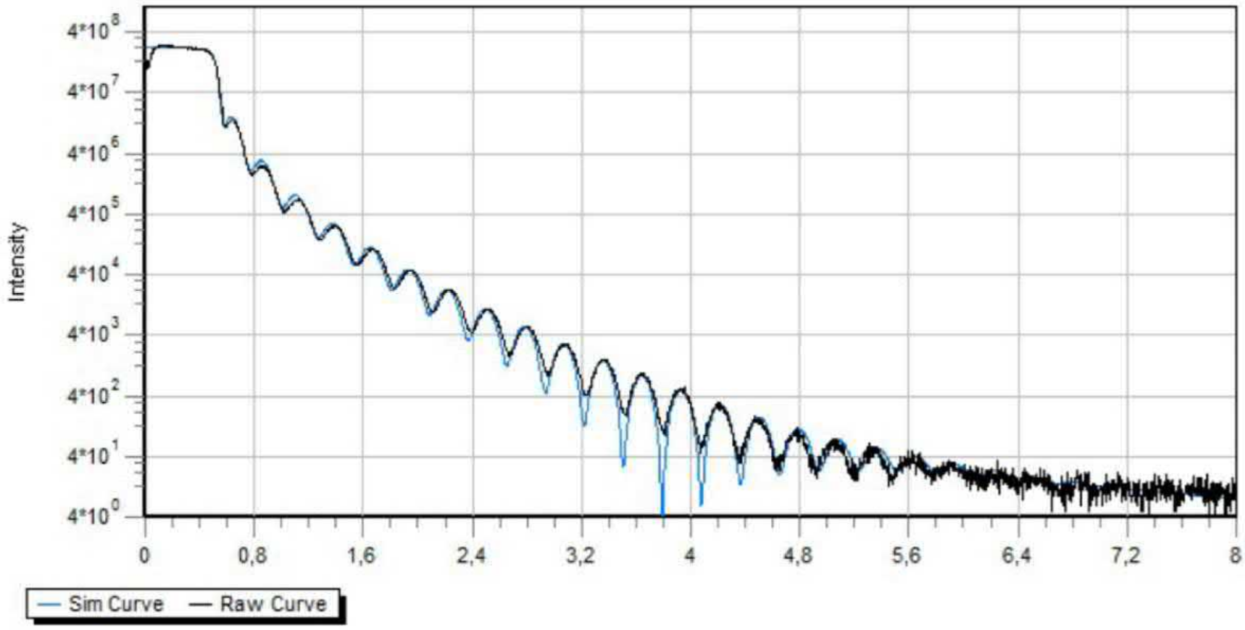

Figure S2: X-ray reflectometry (XRR) curve of Al_2O_3 (black curve) and its fitting curve (blue curve).

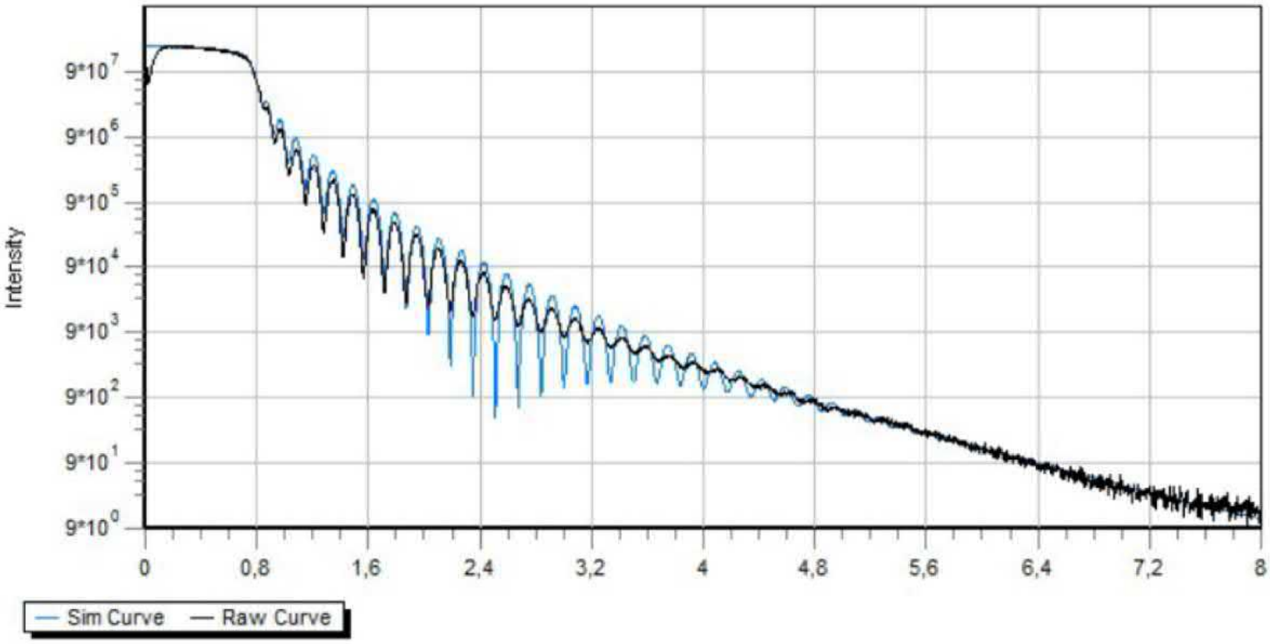

Figure S3: X-ray reflectometry (XRR) curve of HfO_2 (black curve) and its fitting curve (blue curve).

S.3 Focal Plane Intensity

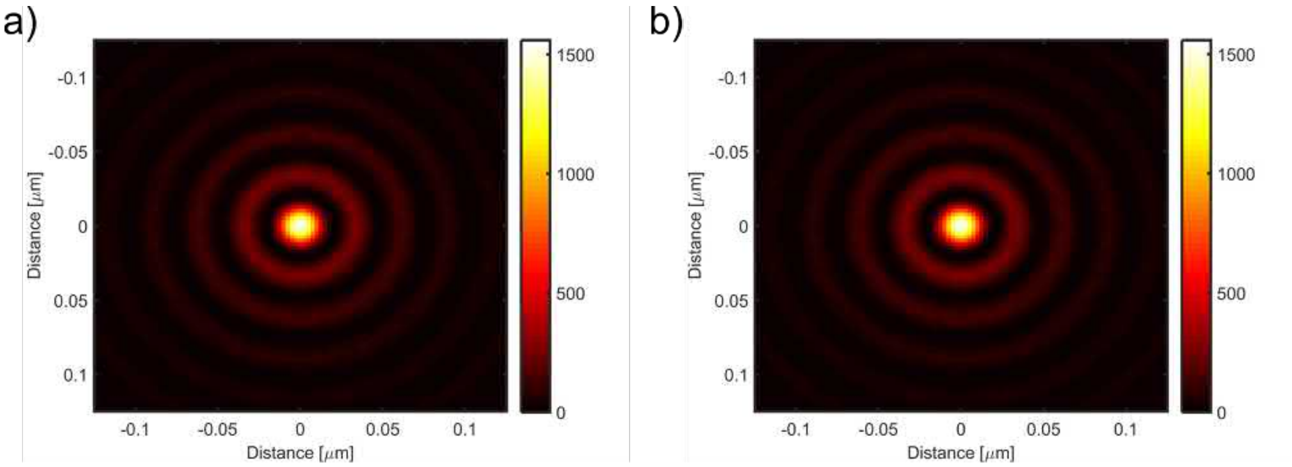

Figure S4: Fourier beam propagation results for an FZP with 30 μm Beamstop diameter and perfect zone positions a) and for an FZP with 31.4 μm Beamstop diameter and 700 nm systematically shifted zones. The change in the intensity is trivial.

S.4 Parasitic deposition of Pt during ion beam induced deposition of the beamstop

Figure S5 shows Pt deposition stages. Pt is generally deposited in four consecutive steps as depicted in Figure S5a-d. The resulting layer is generally rich in C and Ga elements. In this study a mixture of Pt-Ga-C was assumed according to literature (see main text).

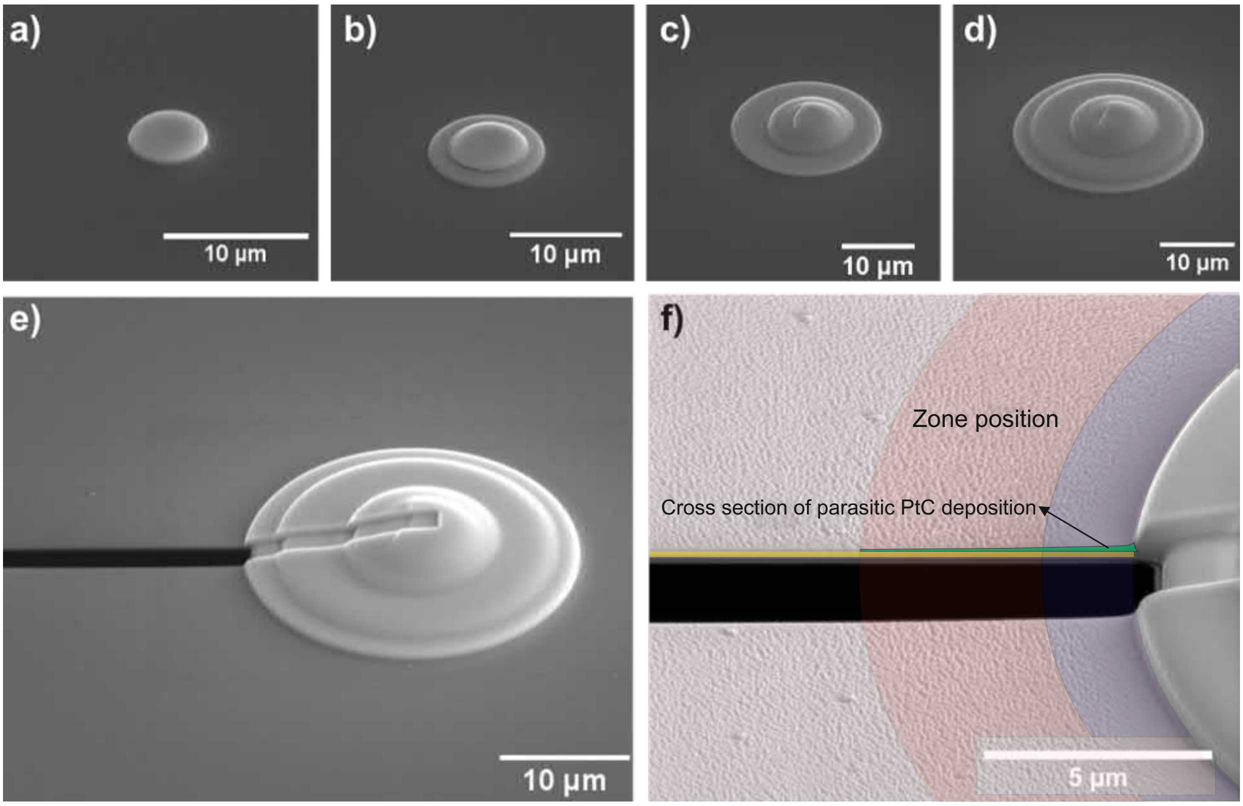

Figure S5: Scanning electron microscopy (SEM) images of ion beam induced Pt-Ga-C test deposition on Si_3N_4 membrane coated with Au showing the significance of parasitic effects during beamstop deposition. The beam stop is usually deposited in 4 consecutive steps at 30 keV accelerating voltage. a) Diameter of d = 5 μm, beam current I = 100 pA b) Diameter of d = 10 μm, beam current of I = 300 pA c) Diameter of d = 20 um, beam current of I = 1 nA d) Diameter of d = 25 μm, beam current of I = 1 nA, e) a cross section in the FIB was milled out to determine the thickness of the Pt-Ga-C layer. f) Cross section image of a higher magnification image of the Pt-Ga-C parasitic deposition. Pink area represents the location of the FZP zones. Green area shows the thickness of the Pt-Ga-C layer. Yellow area shows the deposited Au layer.

S.5 SEM images of PFIB fabricated micropillars

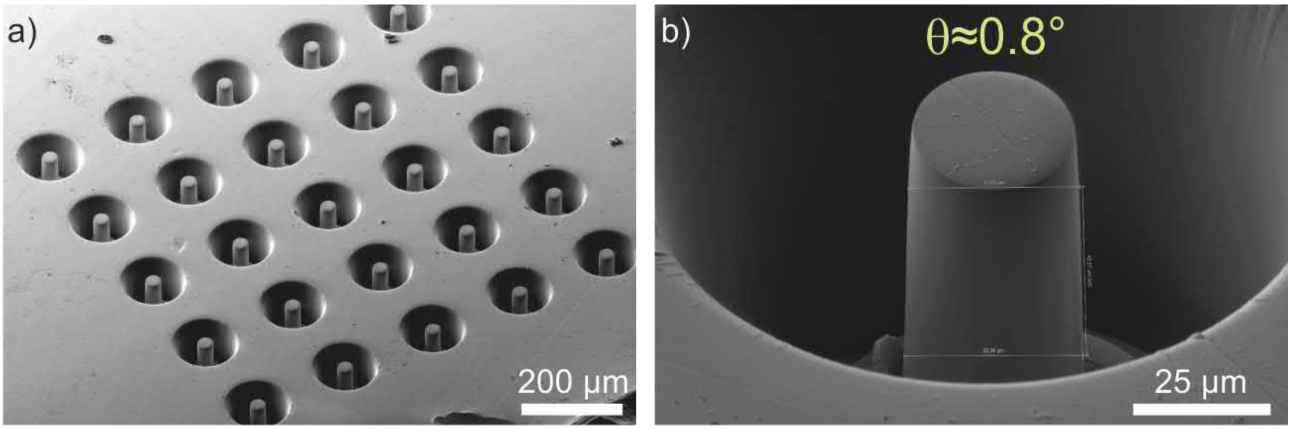

Figure S6: SEM (secondary electron) images of tilted multilayers fabricated on Au (111) substrate *via* PFIB with a tilt angle of $\theta \approx 0.8°$. a) an overview image of array an of micropillars, b) a higher magnification SEM image confirming the quality of the fabricated micropillars.

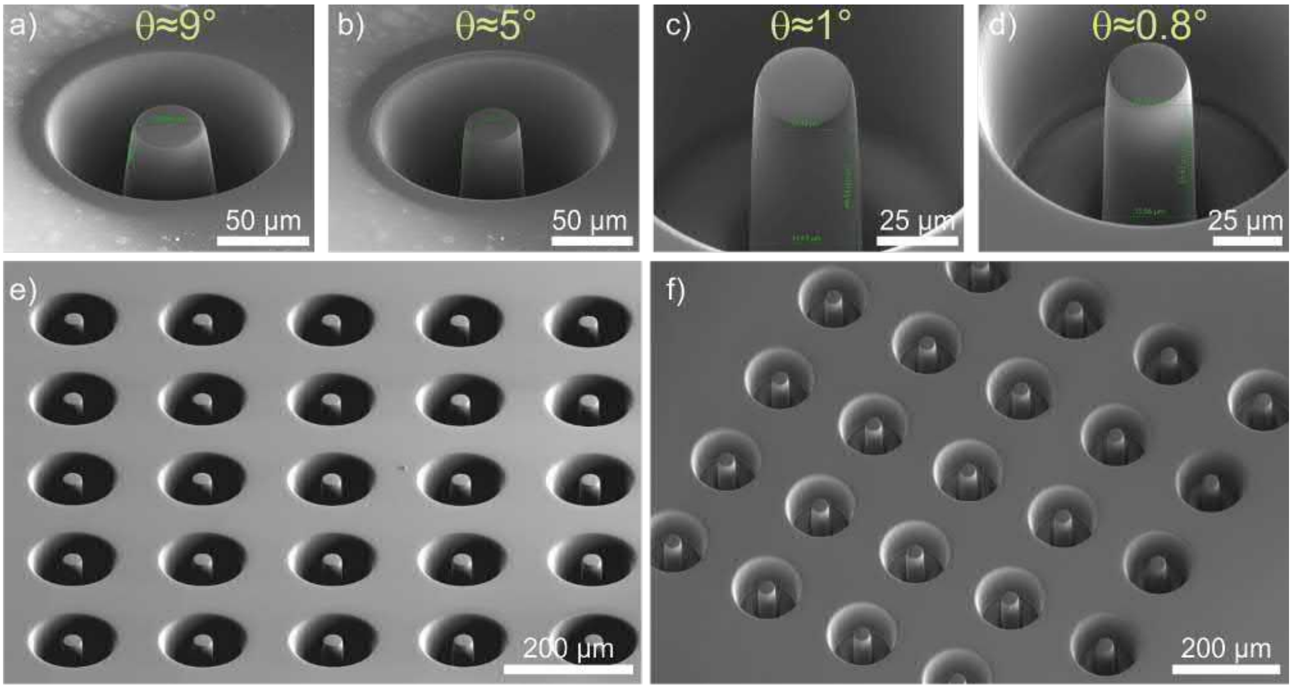

Figure S7: SEM (secondary electron) images of tilted multilayers fabricated on Si (100) substrate *via* PFIB with various tilt angles. a) $\theta \approx 9°$, b) $\theta \approx 5°$, c) $\theta \approx 1°$, d) $\theta \approx 0.8°$, e) an array of $\theta \approx 1°$ tapered micro pillars, f) an array of $\theta \approx 0.8°$ tapered micro pillars.

S.6 Diffraction Efficiency calculations

The thin grating approximation (TGA) as applied to the phase zone plates by Kirz et al.[63] offers a straightforward calculation for estimating the diffraction efficiency of FZPs. However, it relies on the planar diffraction theory, and is accurate only for FZPs with small aspect ratios. It is also independent of the propagation angle of the waves, therefore cannot be used to estimate the effect of tilt angles for high aspect ratio FZPs. For high-resolution FZPs, where the necessary aspect ratios are extremely large, the focusing performance depends strongly on the satisfaction of the Bragg angle.[69] This makes the use of CWT calculations essential. The CWT parameters used in this paper are summarized in the Table below.

Table S1: CWT parameters used for diffraction efficiency calculations. The magnification stands for the ratio of the distance of the source and focal distance. A magnification of 10000 is selected to mimic a plane wave illumination as in the case of our STXM set-up.

	Materials	Density ($g\ cm^{-3}$)	Magnification	Calculated for	Line:Space
Table 1	Al_2O_3/HfO_2	3.0/8.9	10000	Outermost period (Local)	1:2
Fig. 5e-h	Al_2O_3/SiO_2	3.0/8.9	10000	Outermost period (Local)	1:1
Fig. 6a	Al_2O_3/SiO_2	3.0/2.1	10000	Every zone (Integrated)	1:1

S.7 Supporting comment on FIB damage

Ga^+ ion implantation and ion damage during FIB machining are most pronounced under normal incidence ion beam milling. During glancing incidence polishing such as the case in the polishing step described here, Ga^+ ion implantation decreases. While the adverse effects can be significant for TEM samples or plasmonic properties of gold films fabricated via FIB, ML-FZP is less prone to such effects. The damage layer (amorphization + implantation) for 30 kV polishing step can be expected to be roughly 30 nm deep on both surfaces which constitute less than 7 % of the total thickness for ML-FZP with parallel zones and less than 3 % for the tilted ML-FZP. An improvement in this respect can be easily implemented by reducing the accelerating voltage in the final polishing step.[70] In the case of pillar fabrication, one can expect the implanted Ga^+ to be most abundant at the bottom of the trenches rather than pillar walls, both of which serve no functional purpose. Therefore, for the application described here, we conclude that the implanted gallium ions do not constitute a deleterious effect.
